# Cortex *Cercis chinensis* Granules Attenuate *Streptococcus pneumoniae* Virulence by Targeting Pneumolysin

**DOI:** 10.1155/2020/8537026

**Published:** 2020-06-16

**Authors:** Yan Xu, Yanbo Wang, Yinan Guo, Lina Wei, Lizhong Ding, Zhongtian Wang, Liping Sun

**Affiliations:** ^1^Changchun University of Chinese Medicine, Changchun, Jilin 130117, China; ^2^Affiliated Hospital of Changchun University of Chinese Medicine, Changchun, Jilin 130021, China

## Abstract

Pore-forming toxins produced by bacteria are some of the most important molecular weapons for bacterial virulence. Pneumolysin (PLY) is a pore-forming toxin secreted by *Streptococcus pneumoniae* (*S. pneumoniae*) and plays a vital role in the spread, colonization, and invasion of this bacterium in the host, indicating that PLY is a promising target for developing treatments against *S. pneumoniae* infection. In this study, Cortex *Cercis chinensis* granules (CCCGs), a prescription drug on the market, were shown to inhibit the pore-forming activity of PLY and protect against PLY-mediated cell hemolysis and A549 cell death without antibacterial activity or inhibition of PLY production. In addition, CCCG treatment inhibited the oligomerization of PLY. Animal experiments showed that CCCGs can reduce the death of mice infected with *S. pneumoniae*, the degree of pathological damage to the lungs, and the levels of TNF-*α* and IL-6 in the lungs. In summary, our results demonstrated that CCCGs, a marketed Chinese medicine, inhibit PLY activity and subsequently attenuate *S. pneumoniae* virulence, which would offer a novel strategy for fighting *S. pneumoniae* infection and a new use for CCCGs.

## 1. Introduction

Pore-forming toxins (PFTs) produced by bacteria act on the plasma membrane of eukaryotic cells and form a pore structure on the cell membrane, thus disrupting the concentration of liquid inside and outside the cell and causing cell swelling and cell lysis [1]. Pneumolysin (PLY) is such a toxin secreted by *Streptococcus pneumoniae* (*S. pneumoniae*) and is a multifunctional protein consisting of 471 amino acids with a molecular weight of 53 kDa. During the establishment of *S. pneumoniae* infection, PLY in the cytoplasm cannot be directly secreted into the extracellular space. However, after cell wall lysis by autolysin, antibiotic action, or the host-mediated immune response, PLY can be released extracellularly to exert its pore-forming activity [2]. Furthermore, 34–50 PLY monomer molecules can form oligomerized anterior pore complexes and bind to the membrane cholesterol to form a barrel-shaped transmembrane pore of approximately 25 nm in diameter, thereby rupturing the cell [3]. In addition, the release of PLY can also facilitate *S. pneumoniae* evasion of host defenses [4] and trigger acute lung injury and pulmonary fibrosis through direct cytotoxicity and indirect proinflammatory effects [5, 6]. Furthermore, the production of PLY increases the incidence of acute cardiac events [7] and the risk of otitis media [8], meningitis [9], and even death [2]. Given the key role of PLY in the pathogenesis of *S. pneumoniae*-related organ damage and dysfunction caused by invasive pneumococcal disease (IPD), loss of PLY can significantly reduce the number of *S. pneumoniae* adhering to the human body and reduce IPD [10]. Thus, targeting PLY may be a promising anti-infection strategy to treat *S. pneumoniae* infection or improve the efficacy of antibiotics.

Agents directed against this toxin include murine monoclonal antibodies, PLY-4 and PLY-7, which target various epitopes on the toxin, blocking the binding to eukaryotic cell membranes and cytolytic activity [11]. Additionally, *β*-sitosterol [12], apigenin [13], and several other plant-derived agents [14, 15] have been reported to inhibit the oligomerization of PLY monomers. The problem is that none of these antibodies or inhibitors can be used in clinical or even preclinical research. Therefore, we tried to find a new drug that inhibits PLY activity from among Chinese medicine granules, which have been used for thousands of years with effectiveness and safety in bodily experiment verification for long-term use in humans. Our research is an attempt to explore new therapeutic effects or clinical use of traditional Chinese medicine particles from an experimental perspective.

Cortex *Cercis chinensis* (CCC), a Chinese medicine called ZI JINGPI, is the dry root bark of the Magnoliaceae plant *Kadsura longipedunculata* Finet et Gagnep. CCC is derived from “*the Materia Medica of Rihuazi*,” a medical book of the five dynasties of China, which is reported to have the effects of promoting blood circulation and meridian circulation, reducing swelling, and detoxifying and is used for the treatment of irregular menstruation, such as amenorrhea, dysmenorrhea, and bloating, worms, and snake bites. Here, we further found that Cortex *Cercis chinensis* granules (CCCGs), a commercial product of CCC, possess inhibitory effects against PLY activity and *S. pneumoniae* virulence.

## 2. Materials and Methods

### 2.1. Cells and Bacteria

Alveolar epidermal cells (A549) were obtained from the ATCC (Manassas, VA) and cultured in DMEM with 10% fetal bovine serum (FBS, Biological Industries, Israel).

*Streptococcus pneumonia*e strain D39 serotype 2 (NCTC 7466) was kindly provided by Professor Jian Huang from Zunyi Medical University (Zunyi, China) and cultured in the Todd Hewitt broth with 1% yeast extract (THY media) at 37°C with 5% CO_2_.

### 2.2. Cortex *Cercis chinensis* Granules

CCCGs were purchased from Jiangyin Tianjiang Pharmaceutical Co., Ltd., (Jiangyin, China), and the production batch number was 19046614. CCCGs are a traditional Chinese medicine granule prescription drug approved by the China Food and Drug Administration that have been verified to meet the required standard of the China Food and Drug Administration. This drug was dissolved in DMSO for the following use.

### 2.3. Antibacterial Activity Determination

The minimum inhibitory concentration of CCCGs against the *S. pneumoniae* strain D39 was examined according to a previous study [16]. The growth of the *S. pneumoniae* strain D39 treated with various concentrations (0, 4, 8, 16, or 32 *μ*g/ml) of CCCGs was determined by monitoring the OD600 nm of each sample every 30 min.

### 2.4. Recombinant Pneumolysin Preparation

Construction of a pET28a vector containing the *ply* sequence and the PLY protein purification methods were based on previous descriptions [17]. In short, harvested cells were lysed by sonication, and the supernatant of the centrifuged cell lysate was loaded onto a Ni-NTA agarose column. The target protein was flushed with an elution buffer (PBS containing 200 mM imidazole, pH 7.4). Then, the recombinant protein was concentrated using a Millipore Amicon filter (30 kDa cutoff) for desalting.

### 2.5. Hemolysis Assay

Here, 1.0 *μ*l of purified PLY (0.2 mg/ml) in 965 *μ*l of PBS was treated with various concentrations (0, 4, 8, 16, or 32 *μ*g/ml) of CCCGs at 37°C for 30 min. Then, 25 *μ*l of defibrinated sheep red blood cells were added to the system and incubated at 37°C for 10 min. Finally, after centrifugation at 3000 ×g for 5 min, 200 *μ*l of the supernatant was pipetted to determine the hemolytic activity at OD_543 nm_. The sample treated with DMSO was used as 0 *μ*g/ml CCCGs.

### 2.6. Immunoblot Analysis

*S. pneumoniae* D39 cells were grown with different concentrations of CCCGs (0, 4, 8, 16, and/or 32 *μ*g/ml). An equal amount of bacterial culture supernatant from each sample was suspended in the same volume of the Laemmli sample buffer, boiled for 10 min, and separated by 12% SDS-PAGE. Following transfer onto a PVDF membrane, the PLY protein was detected using a monoclonal antibody against PLY (1 : 1000, Abcam, Cambridge, UK) and HRP-conjugated secondary anti-mouse antibodies (1 : 2000, Proteintech) and developed with the ECL reagent (Thermo Scientific, Rockford, IL, USA).

### 2.7. Pneumolysin Oligomerization Analysis

Different concentrations of CCCGs (0, 4, 8, 16, and/or 32 *μ*g/ml) were mixed with PLY at 37°C for 1 h. Then, 5x SDS-PAGE loading buffer without *β*-mercaptoethanol was added and incubated for 10 min at 50°C. Twenty microliters of the samples were separated by 6% SDS-PAGE and transferred onto a PVDF membrane. Then, the monomers or oligomers were detected using immunoblot analysis as described above. The sample treated with DMSO was used as 0 *μ*g/ml CCCGs. The grey scales of the bands for PLY oligomers/monomers were analysed using Image-Pro Plus software.

### 2.8. Green/Red and Cytotoxicity Test

A549 cells were trypsinized and seeded at a density of 2 × 10^4^ cells per well in 96-well plates for the overnight culture. The cells were incubated for 5 h with PLY that was treated with or without CCCGs in a 37°C incubator. Cell viability was observed using live/dead (green/red) reagents (Invitrogen, Carlsbad, CA, USA) according to the manufacturer's instructions. Finally, images were acquired using a confocal laser scanning microscope (Olympus, Tokyo, Japan). A cytotoxicity detection kit (Roche, Mannheim, Germany) was used for the determination of lactate dehydrogenase (LDH) activity. Following centrifugation in a 96-well plate (1000 rpm, 10 min), the supernatant was mixed with the reaction reagents for incubation for 30 min in the dark. Then, LDH activity was measured at OD_492 nm_ by a microplate reader (Tecan, Salzburg, Austria). The sample treated with DMSO was used as 0 *μ*g/ml CCCGs.

### 2.9. Mouse Infection Assays

BALB/c mice (female, 6–8 weeks old, 20–22 g) were purchased from Liaoning Changsheng Biotechnology Co., Ltd., (Shenyang, China) and maintained in accordance with the NIH Guide for the Care and Use of Laboratory Animals. All mouse experiments were approved by the Ethics Committee of the Changchun University of Chinese Medicine. The *S. pneumoniae* strain D39 was cultured in THB at 37°C until the OD_600 nm_ reached 0.4 (midlogarithmic phase), collected by centrifugation (1000 rpm for 10 min), and washed three times with PBS. Except for those in the healthy control group, each mouse was nasally infected with 1.5 × 10^8^ colony-forming units (CFUs) of bacteria. These mice were subcutaneously injected with CCCGs (40 mg/kg) or DMSO every 8 h, and the mice were observed for survival for 72 h (*n* = 12). For other mouse infection assays (*n* = 8), the lungs from sacrificed mice were used for gross pathological analysis by a camera or histopathological analysis by hematoxylin-eosin staining under light microscopy at 48 h after infection. Bronchoalveolar lavage fluid was collected in two bronchial sterile PBS infusions, totaling 500 *μ*l. After liquid centrifugation, TNF-*α* and IL-6 levels were measured according to the instructions of an ELISA kit (eBioscience, San Diego, CA, USA).

### 2.10. Statistical Analysis

The data were analyzed by GraphPad Prism 6.0 (GraphPad Software) using Student's *t*-test; ^*∗*^*p* < 0.05 and ^*∗∗*^*p* < 0.01, and the data are expressed as mean ± SD (*n* ≥ 3).

## 3. Results

### 3.1. CCCGs Inhibit PLY-Mediated Hemolysis

**Figure 1:**
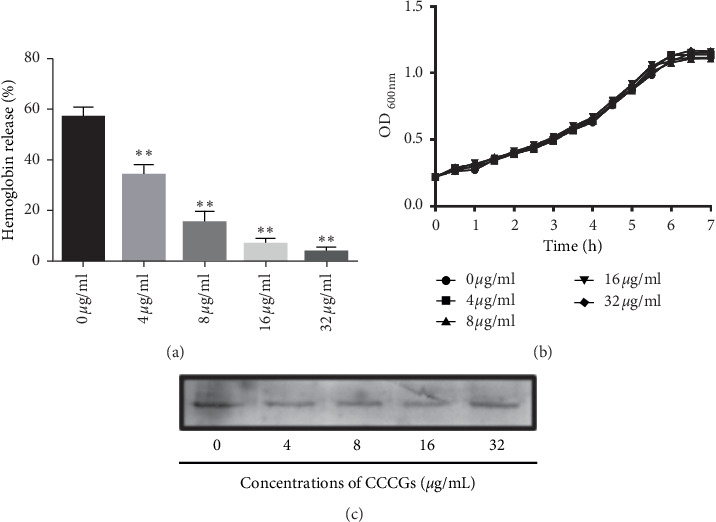
CCCGs inhibit PLY activity without affecting *S. pneumoniae* viability or PLY production. (a) PLY was pretreated with the indicated concentrations of CCCGs, and the activity of PLY was tested using a hemolysis assay. Data are presented as the mean ± SD (*n* = 3). ^*∗∗*^*p* < 0.01. (b) *S. pneumoniae* was cocultured with various concentrations of CCCGs, and then the OD600 nm of each sample was determined every 30 min. (c) *S. pneumoniae* was cocultured with various concentrations of CCCGs, and the PLY production in the bacterial culture supernatants was examined by immunoblot analysis.

We first performed a hemolysis test to screen for potential PLY inhibitors and found that CCCG treatment reduced PLY-mediated red blood cell hemolysis in a concentration-dependent manner, as shown in Figure 1(a). These results proved that CCCGs block the activity of PLY.

### 3.2. CCCGs Do Not Inhibit the Growth of *S. pneumoniae* or the Production of PLY

Next, we mapped the antibacterial profile of CCCGs. As shown in Figure 1(b), CCCGs did not directly inhibit the growth of *S. pneumoniae* at concentrations of 4, 8, 16, or 32 *μ*g/ml. In addition, the minimum inhibitory concentration (MIC) of CCCGs against *S. pneumoniae* was no less than 512 *μ*g/ml. Then, we continued to verify whether CCCGs would affect PLY production in *S. pneumoniae*, and as shown in Figure 1(c), CCCGs had no effect on PLY production at every concentration (up to 32 *μ*g/ml) tested in this study. In summary, our results demonstrated that CCCGs can prevent PLY-mediated cell hemolysis without affecting the growth of *S. pneumoniae* or PLY production.

### 3.3. CCCGs Inhibit PLY Oligomerization

**Figure 2:**
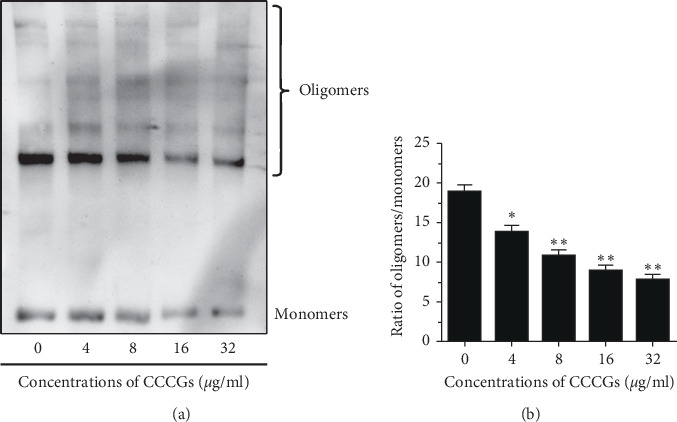
CCCGs reduce the oligomerization of PLY. (a) PLY was pretreated with the indicated concentrations of CCCGs, and monomers and oligomers were determined using immunoblot analysis. (b) The grey scales of the bands for PLY oligomers/monomers were analysed using Image-Pro Plus software. ^*∗*^*p* < 0.05; ^*∗∗*^*p* < 0.01.

The above results suggested that CCCG treatment could directly inhibit the activity of PLY; however, the potential mechanism of such inhibition was not clear. Pore-forming activity is the main physiological function of PLY, which is closely associated with the oligomerization of PLY. Thus, we speculated that CCCGs achieve an inhibitory function by blocking the oligomerization of PLY. Western blot experiments confirmed our conjecture. As shown in Figure 2, CCCGs inhibited the oligomerization of PLY, thereby reducing the activity of PLY.

### 3.4. CCCGs Inhibit PLY-Mediated Death of A549 Cells

**Figure 3:**
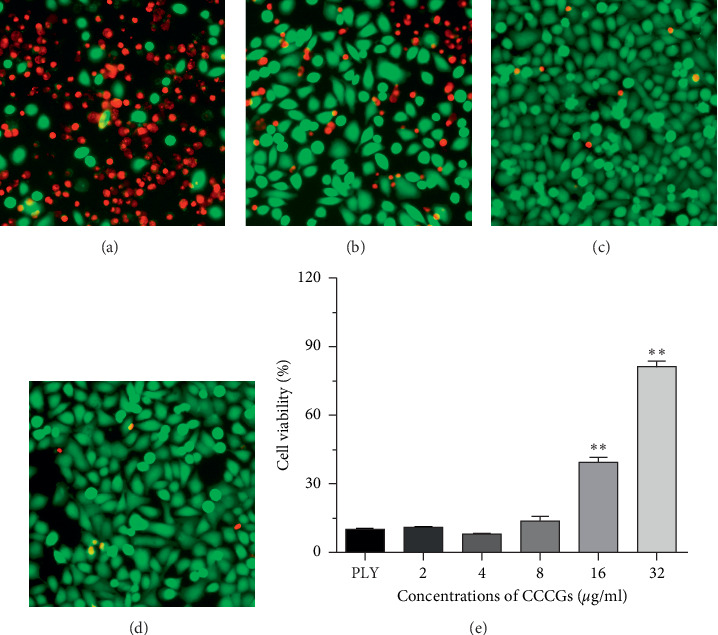
CCCGs attenuate PLY-mediated cytotoxicity. A549 cells were incubated for 5 h with PLY that was treated with or without CCCGs. Then, the cells were stained with live (green) or dead (red) reagent and observed under a confocal laser scanning microscope. (a) A549 cells cocultured with PLY; (b) A549 cells cocultured with PLY in the presence of 4 *μ*g/ml CCCGs; (c) A549 cells cocultured with PLY in the presence of 32 *μ*g/ml CCCGs; and (d) A549 cells cocultured with 32 *μ*g/ml CCCGs. (e) A549 cells were incubated for 5 h with PLY that was treated with or without CCCGs, and the LDH released into the supernatants of the coculture system was determined using a cytotoxicity detection kit. Data are presented as mean ± SD (*n* = 3). ^*∗∗*^*p* < 0.01.

To determine whether CCCGs can inhibit the cytotoxicity of PLY at the cellular level, A549 human alveolar basal epithelial cells were selected for the following assays. After incubation with PLY that was treated with or without CCCGs, the cytotoxicity of A549 cells was observed. As shown in Figure 3(a), PLY incubation led to robust cell death, as evidenced by the fact that most cells were stained red. However, such red cells were significantly reduced following CCCG treatment (Figures 3(b) and 3(c)). Additionally, CCCG treatment exhibited no cytotoxicity against A549 cells (Figure 3(d)). In agreement with this observation, the cell viability was significantly increased for PLY-treated A549 cells in the presence of increasing concentrations of CCCGs (Figure 3(e)). Taken together, our results suggested that CCCG treatment provides robust protection against PLY-mediated cytotoxicity in A549 cells.

### 3.5. CCCGs Protect Mice from *S. pneumoniae* Infection

**Figure 4:**
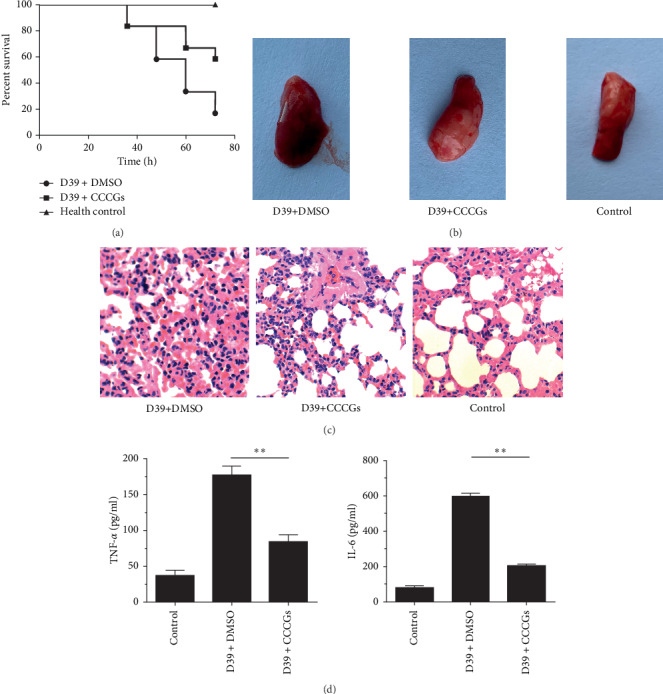
CCCGs protect mice from *S. pneumoniae* infection. BALB/c mice were nasally infected with 1.5 × 10^8^ CFUs of *S. pneumoniae* and treated with 40 mg/kg CCCGs or DMSO for 72 h. (a) The survival of infected mice with the indicated treatment was monitored for 72 h. The lungs from sacrificed mice were used for gross pathological analysis by (b) a camera or (c) histopathological analysis by hematoxylin-eosin staining under light microscopy at 48 h after infection. (d) The inflammatory factors (TNF-*α* and IL-6) in the bronchoalveolar lavage fluid were examined by ELISA. Data are presented as mean ± SD (*n* = 3). ^*∗∗*^*p* < 0.01.

The *in vitro* inhibition of PLY activity by CCCGs prompted us to examine whether protection occurs *in vivo*. BALB/c mice were selected for animal experiment verification. After nasal infection of mice with *S. pneumoniae*, we found that CCCG treatment could protect mice from *S. pneumoniae*-mediated death (Figure 4(a)), as evidenced by 16.67% survival and 58.33% survival for infected mice that received DMSO and CCCG, respectively. Moreover, compared with that in the lungs of the *S. pneumoniae* D39 infection group, the pathological damage in the lungs of the CCCG treatment group was significantly alleviated, with more ruddy and shiny appearance (Figure 4(b)). The pathological lung sections showed that the lung inflammation of mice was significantly reduced after CCCG treatment (Figure 4(b)), which is consistent with the results in Figure 4(b). Finally, we measured the levels of TNF-*α* and IL-6 in bronchoalveolar lavage fluid in infected mice. As shown in Figure 4(d), CCCG treatment also reduced the amount of proinflammatory factors in the lungs of *S. pneumoniae*-infected mice. Taken together, our results established that CCCGs protected against *S. pneumoniae* infection in mice by inhibiting the pore-forming activity of PLY.

## 4. Discussion

PFTs are complex and widespread virulence factors produced by pathogenic bacteria that can destroy the host's epithelial barrier, kill immune cells, and facilitate bacterial spread and multiplication [18]. Similar to most PFTs, PLY directly causes cell lysis at high concentrations and induces cell inflammation and apoptosis at low concentrations [19]. After the cell membrane interacts with a low concentration of PLY, cells can even self-repair the membrane by, for example, lysosomal decomposition after endocytosis [20] or directly discarding the pores from the cell [21], ultimately avoiding death. However, regardless of whether PLY can cause cell death, this toxin can greatly affect cell function, which is important for *S. pneumoniae* virulence. However, inhibition of PLY production may prompt *S. pneumoniae* to rapidly mutate to find opportunities for survival in the complex host internal environment. This “human-induced evolution” approach will instead stimulate *S. pneumoniae* to produce other invasins and virulence mechanisms, ultimately leading to human failure in this conflict [22]. Thus, direct neutralization of PLY activity instead of preventing its production or directly killing the bacteria becomes an ideal choice.

CCCGs have been reported to possess anti-inflammatory and bactericidal effects in ancient Chinese medical books. Here, we aimed to find a unique antibacterial drug from the treasure trove of the civilization of the Chinese nation, as Tu did [23]. On the other hand, CCCGs are a drug developed by good manufacturing practice (GMP) and have been used in hospitals and communities in China for many years. They have strict production processes from raw materials, decoction pieces, creams, intermediates, and finished products, with the production batch number listed in the materials and methods.

In this study, CCCGs were confirmed to have no direct antibacterial effect against *S. pneumoniae* at concentrations up to 32 *μ*g/ml. However, CCCG treatment significantly reduced PLY-mediated cytotoxicity through hemolysis experiments and live/dead cell experiments. Western blot analysis further demonstrated that CCCGs can neutralize PLY pore-forming activity without affecting PLY production by *S. pneumoniae*. Animal experiments further verified that CCCGs can protect mice infected with *S. pneumoniae* from death and significantly reduce lung inflammation and the release of proinflammatory factors. In short, all these experiments demonstrated that CCCGs can reduce the pathogenicity of *S. pneumoniae* by neutralizing the cytotoxicity of PLY. This anti-infection strategy by CCCGs does not put evolutionary pressure on *S. pneumoniae*.

Human technology and antibacterial strategies have continued to evolve, but this long-term enemy has not been completely eliminated. Antibacterial infection strategies should maintain a balance between bacteria and hosts but not employ a simple bactericidal action. As reported in our study, CCCGs are such a drug to fight bacterial infection without putting selective pressure on the targeted bacteria. The use of plants and their extracts to treat diseases is not a unique method only in China. Similar methods are available in India, South Korea, Japan, most African regions, and the Amazon. Research on plant-based antibacterial drugs may be the key research direction of supplementing and replacing antibiotics in the future.

In short, our study successfully used CCCGs as a blocker of PLY pore-forming activity and subsequently reduced the pathogenicity of *S. pneumoniae*, which demonstrated the potential of Chinese medicine to treat bacterial infection.

## Data Availability

The experimental data used to support the findings of this study are included within the article.
